# Structure, dynamics and stability of water/scCO_2_/mineral interfaces from *ab initio* molecular dynamics simulations

**DOI:** 10.1038/srep14857

**Published:** 2015-10-12

**Authors:** Mal-Soon Lee, B. Peter McGrail, Roger Rousseau, Vassiliki-Alexandra Glezakou

**Affiliations:** 1 Fundamental and Computational Sciences Directorate, Pacific Northwest National Laboratory, Richland, WA 99352; 2 Energy and Environment Directorate, Pacific Northwest National Laboratory, Richland, WA 99352.

## Abstract

The boundary layer at solid-liquid interfaces is a unique reaction environment that poses significant scientific challenges to characterize and understand by experimentation alone. Using *ab initio* molecular dynamics (AIMD) methods, we report on the structure and dynamics of boundary layer formation, cation mobilization and carbonation under geologic carbon sequestration scenarios (T = 323 K and P = 90 bar) on a prototypical anorthite (001) surface. At low coverage, water film formation is enthalpically favored, but entropically hindered. Simulated adsorption isotherms show that a water monolayer will form even at the low water concentrations of water-saturated scCO_2_. Carbonation reactions readily occur at electron-rich terminal Oxygen sites adjacent to cation vacancies that readily form in the presence of a water monolayer. These results point to a carbonation mechanism that does not require prior carbonic acid formation in the bulk liquid. This work also highlights the modern capabilities of theoretical methods to address structure and reactivity at interfaces of high chemical complexity.

A large number of important chemical phenomena happen at solid-liquid interfaces relevant to geochemistry^1^,^2^,^3^,^4^, atmospheric phenomena^5^ and catalysis^6^,^7^,^8^,^9^. Critical scientific questions arise regarding the species present at the boundary layer, the impact of the dynamics and structure of this layer on surface reconstruction, reactivity and adsorbed molecular states. Unlike solid-gas interfaces, solid liquid interfaces are buried in condensed media and pose a stringent challenge to characterize by experimentation alone. Advances in computational methods have afforded us with the unique opportunity to address these issues at the molecular level at different length and time scales. Classical molecular dynamics (MD) has been used as a tool to understand the thermodynamics of interfaces and surface adsorption^10^,^11^,^12^,^13^,^14^. These methods although extremely valuable, are highly reliant on predetermined intermolecular potentials, which may not necessarily be transferrable from the bulk liquid to the interfacial environments, or able to reproduce surface reactivity. *Ab initio* molecular dynamics (AIMD)^15^ on the other hand, is in principle more transferrable, but is more limited in its ability to represent models of the requisite chemical complexity, size and time scale. Nevertheless, recent advances in linear scaling density functional theory methods have allowed us to approach realistic interfacial models^16^,^17^,^18^. This work addresses surface adsorption and reactivity in a prototypical mineral/water/scCO_2_ system, in the context of carbon management, with strong implications for CO_2_ sequestration strategies and general interfacial phenomena.

Feldspars are abundant in cap rock formations targeted for sequestration where permanent CO_2_ trapping in the form of carbonates is aided by the presence and dissolution of cations such as Na^+^, K^+^ or Ca^2+^ 19,20. Anorthite, the Ca-end member of the feldspar group, is present in many geologic formations and is of particular interest because of fast dissolution that may significantly affect cap rock stability and porosity^21^,^22^. However, the higher charge cations in anorthite carry the potential of more efficient CO_2_ sequestration and carbonate precipitation. Recent advances in experimental instrumentation, for example X-ray scattering and reflectivity studies^22^,^23^,^24^,^25^ have allowed the unprecedented *in situ* characterization of mineral-water interactions. In addition, surface microanalysis techniques have allowed a closer view of the local structure and concomitant changes at the interface of feldspar minerals with water under different conditions and different compositions^25^. While these studies provide a unique dataset to benchmark our ability to represent and quantify structure, dynamics and reactivity in these complex systems via simulation, they cannot reveal the intimate details of molecular level reactivity. When in contact with minerals, the water response is often film assembly at the interface with various degrees of structure, e.g. ice-like layers^26^ or liquid boundary layers^25^, controlled by hydrophilic or hydrophobic effects^27^,^28^. Remarkably, the structural effects have significant impact in the structure of water or other liquids away from the immediate vicinity of the interface^29^,^30^,^31^,^32^. Reactivity in water-bearing scCO_2_ has received scant attention, in spite of unique mechanistic details of corrosion^33^,^34^ and mineralization^35^ reactions occurring under sequestration conditions.

The relative energetics of water and CO_2_ adsorption as well as their impact on surface reconstruction, cation mobilization and carbonate formation are discussed in terms of enthalpic and entropic contributions. These insights are used to understand the structure and dynamics of the scCO_2_ mineral interface as a function of hydration state and temperature effects. We show that Ca dissolution is enthalpically favorable in the presence of just a water monolayer. Carbonate formation adjacent to cation vacancies is shown to be facile by direct activation of CO_2_, even on the picosecond time scale of this study. Finally, the free energy profile for water adsorption and monolayer nucleation and growth is used to de-convolute the role of energy and entropy in water film formation in the presence of wet scCO_2_.

## Results

### Energetics of H_2_O and CO_2_ adsorption

**Figure 1:**
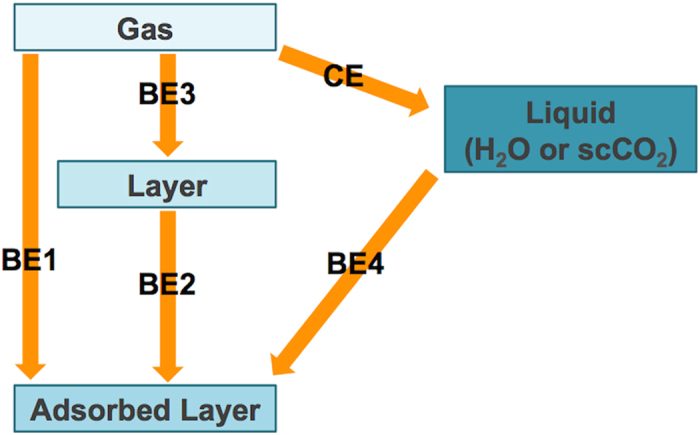
Reaction scheme. Thermodynamic cycle connecting the energy changes of reactions Equations (2)–(6).

Here, we consider the energetics of water and carbon dioxide adsorption by examining the first layer of molecules adjacent to the Ca-rich anorthite surface as extracted from the AIMD simulations with water and scCO_2_ molecules over the mineral, respectively. The extracted structures are optimized to obtain the structure of a stand-alone monolayer of H_2_O and CO_2_, see Supplementary Figs 1 and 2 Table 1, we summarize the adsorption energies per molecule at full-monolayer coverage on the Ca-rich face of the anorthite (001) surface and compare to single-molecule adsorption. For the estimation of the energies, the following formulae were used:


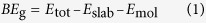



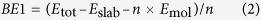



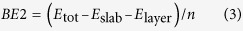



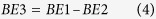



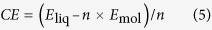



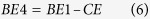


where *E*_mol_ is the energy of gas phase molecule, *E*_tot_ is the total energy of any combined system, *E*_slab_ is the energy for the slab, *E*_layer_/*E*_liq_ is the energy of the H_2_O or CO_2_ layer, or a box of water or scCO_2_, *CE* is the cohesion energy, and *n* is the number of molecules in the simulation. Equation (1) estimates the binding energy of a single, gas-phase molecule to the surface. Equation (2) provides an estimate of the average binding energy to the surface per H_2_O or CO_2_ molecule, including both molecule/surface and molecule/molecule interactions. To de-convolute these energy terms, we introduce equations (3) and (4), where the former provides estimates of the average layer-surface interaction energy, while the latter provides the average molecule-molecule interaction within the layer. Equation (5) estimates the cohesion energy in the pure liquids to juxtapose with the binding energy of the layer to the surface. Equation (6), gives *BE*4, the difference between *BE*1 and *CE*, which is a measure of the energetic preference of a molecule being adsorbed on the surface, as opposed to being solvated in the pure liquid phase. Figure 1 describes how the above quantities are related in a thermodynamic cycle.

Based on the computed values of adsorption energies, the following conclusions can be drawn regarding water film formation. When a full monolayer of water is formed on the surface, the adsorption energy (−97 kJ/mol) only mildly decreases compared to the adsorption of a monomer (−103 kJ/mol). This is a result of strong H_2_O-surface interactions (−82 kJ/mol) and comparatively weak H_2_O-H_2_O interactions within the layer (−15 kJ/mol). The surface-H_2_O interaction can be mainly attributed to the 8 coordination bonds between the 4 top-most Ca cations and H_2_O. These interactions are very similar in magnitude to the hydration energy of the Ca (H_2_O)_6_^2+^ species^36^, which provides an average energy per Ca-water coordination bond of 164 kJ/mol. Using this quantity as guideline, we extrapolate that 8 Ca-H_2_O bonds provide an estimated −93 kJ/mol each. This is slightly higher than the computed quantity BE2, and suggests that the surface Ca-H_2_O interactions are weaker than those in the fully-solvated Ca^2+^. Similarly, the cohesion energy CE of bulk water (−57 kJ/mol) arises from approximately 3.5 H-bonds per H_2_O molecule, averaging −16.3 kJ/mol each.

In the case of CO_2_, the average binding energy of a CO_2_ in the monolayer is lower (−17 kJ/mol) than that of a single CO_2_ molecule (−34  J/mol). More specifically, while the Ca-O_CO2_^17^, or O_surf_-C_CO2_ interactions have a stabilizing effect^37^,^38^, the OH-O_CO2_ interactions are only mildly stabilizing^39^. Thus, CO_2_-CO_2_ interactions in the monolayer provide only a small stabilization of −4 kJ/mol and the CO_2_ layer is only weakly bound. Overall, there is a stronger enthalpic driving force for H_2_O adsorption on the mineral surface than the appreciably weaker one for the CO_2_, suggesting that in carbon sequestration scenarios, water saturated CO_2_ will likely undergo preferential segregation, with H_2_O gravitating towards the surface forming a water film. However, as we will see shortly, entropic considerations may radically influence the speciation at the interface.

### Finite Temperature Effects

**Figure 2:**
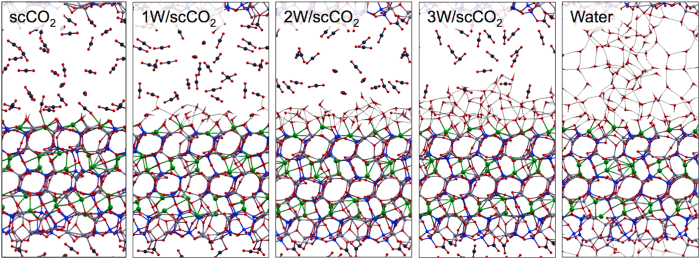
Structures at 323 K. Snapshot of liquid phase structures obtained from AIMD simulations for scCO_2_/H_2_O mixture on anorthite surface. Hydrogen (white), Carbon (dark grey), Oxygen (red), Silicon (blue), Aluminum (silver), Calcium (green).

We assess the impact of the hydration level on mineral dissolution and carbonate formation. For the analysis, we consider the progression of systems ranging from: pure scCO_2_, scCO_2_ with one, two and three water layers, and pure water, snapshots shown in Fig. 2. A summary of cell parameters and simulation times for all the systems can be found in Supplementary Table 1.

**Figure 3:**
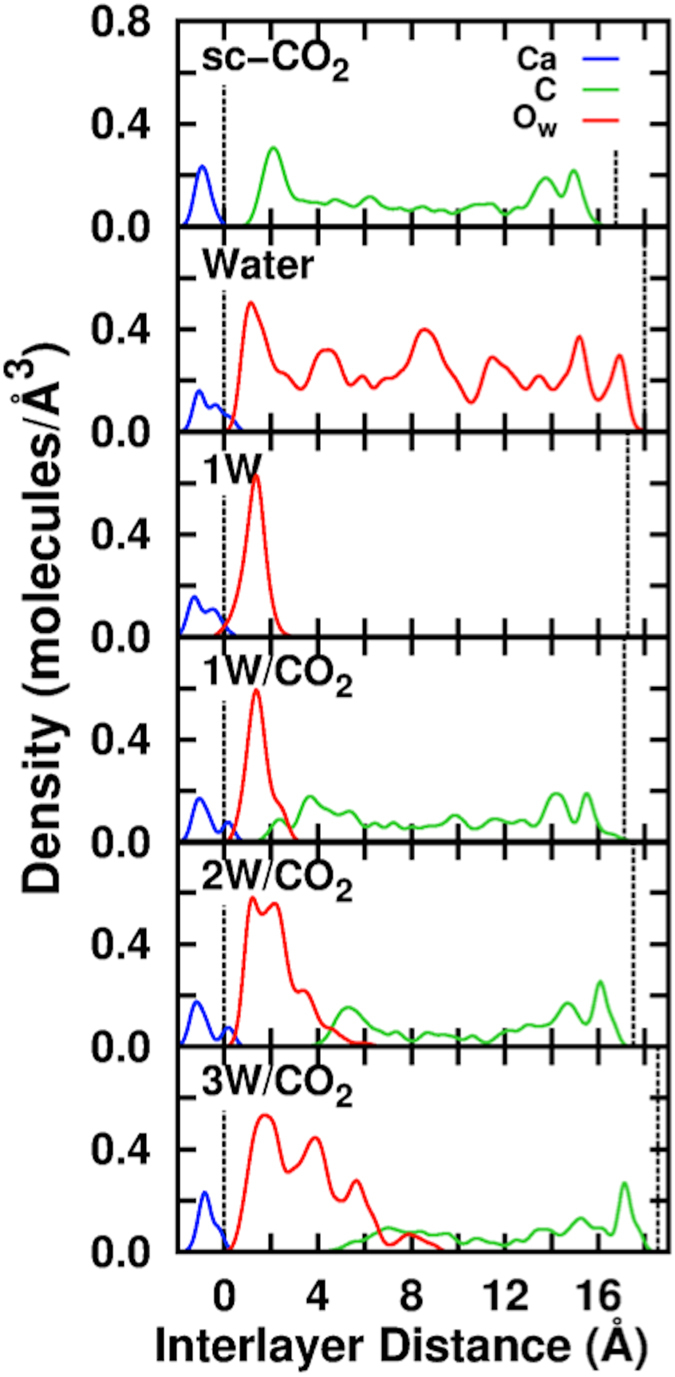
Molecular Densities from AIMD Trajectories. Molecular distributions between the mineral surfaces along *Z*-direction. Red (O of water), Green (C of CO_2_), and Blue (Ca). The dotted lines mark the mineral surface boundaries of the periodic simulation cell.

In Fig. 3, we show the molecular density profiles of Ca, H_2_O and CO_2_ along the direction of the surface normal. The top two panels show the distribution of CO_2_ (sc-CO_2_) and H_2_O (Water) for the surface/pure liquid systems. For pure scCO_2_, we observe a liquid-like layer (calculated density ~ 0.6 g/ml) that is consistent with a super-critical CO_2_ medium. Higher density peaks, at ~2 Å from the surface, are indicative of a denser CO_2_ layer, also observed in the case of other minerals^38^. The bottom 4 panels in Fig. 3 show distinct water layer features with or without scCO_2_. At the H_2_O/scCO_2_ interface, we still observe a higher density CO_2_ layer, however it is less ordered compared to the pure scCO_2_ system. In all cases, diffusion in and out of the CO_2_ layer is evident on the ps time scale. On the Ca-deficient surface, within 5 ps of simulation, formation of carbonates (CO_3_^2−^) is observed from reaction of CO_2_ with surface oxygens O_2_^−^ adjacent to Ca^2+^ vacancies. Conversely, on the Ca-rich face, neither carbonate formation nor strong Ca^2+^/CO_2_ interactions are observed on the simulation time scale. However, as we will discuss later, carbonates can be formed when Ca vacancies occur near the surface.

The water-only system (Water) shows a striation of the molecular density, with a pronounced maximum adjacent to the surface. The water monolayer (1W) is stable at finite temperature and perturbed only slightly by the presence of the scCO_2_ component (1W/CO_2_). Within this layer, water exhibits a diffusion coefficient *D *~ 10^−6^ cm^2^/s only an order of magnitude smaller than water self-diffusion in water (22 × 10^−6^ cm^2^/s)^40^, indicating a mobile water layer. Although water molecules diffuse within this layer, they do not diffuse out of it. Within the layer, each H_2_O is bound to an average of 1.4/1.4 (w/wo scCO_2_) waters and 1.0/1.0 surface oxygens via hydrogen bonds. Each Ca^2+^ is bound to an average of 1.5 water molecules through direct Ca-O_w_ coordination bonds. Compared to the 1W system, the water coordination environment changes in the 2W and 3W systems. Within the first layer, each H_2_O is bound to an average of 2.0/2.0/2.0 (2W/3W/Water systems) waters and 0.2/0.1/0.2 surface oxygens via hydrogen bonds. Each Ca^2+^ is bound to an average of 1.6/1.1/1.1 waters through Ca-O_surf_ bonds. The 2^nd^ and 3^rd^ water layers show a distinctly different intermolecular structure, very similar to that of the bulk water. For instance, the 2^nd^ water layer shows a broader density maxima than the first, even exhibiting a double peak in the 2W system similar to other studies^11^. The 3^rd^ water layer shows progressively lower density maxima, indicating that a finite percentage of waters, ca 2%, reside in a 4^th^ layer. Diffusion coefficients and densities for all these systems are summarized in Supplementary Table 2.

**Figure 4:**
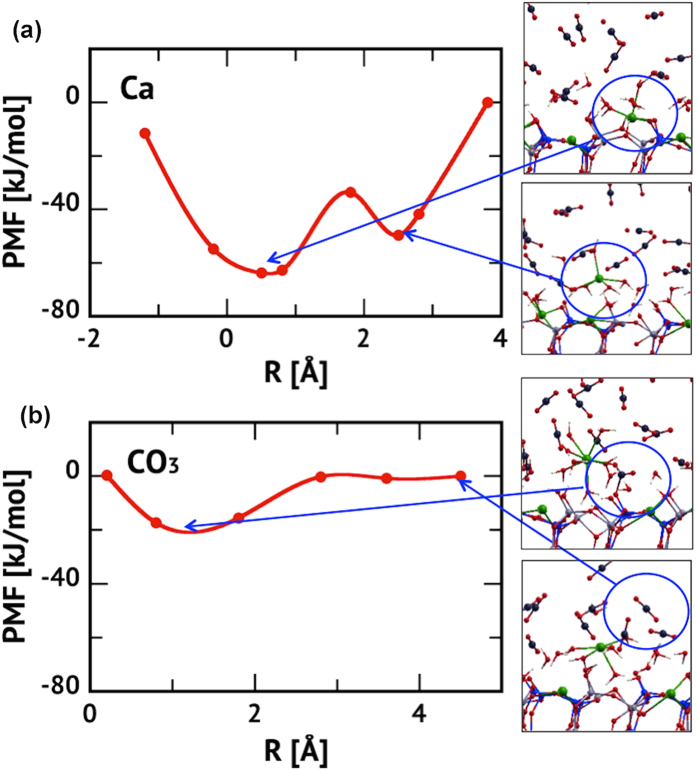
Computed Potential of Mean Force (PMF). (**a**) PMF for Ca dissolution. **(b**) Subsequent carbonation at the newly formed Ca vacancy.

The presence of a water layer, adjacent to the surface, induces large disorder in the Ca positions such that these atoms exhibit finite populations up to 1 Å above their normal lattice positions, see Fig. 3. To examine this further, we performed a simulation with a single Ca atom extracted from the surface into the first water layer of the 1W system. The resulting structure (Fig. 4a and Supplementary Fig. 4) consists of a water-solvated Ca^2+^ species within the 1W film as an outer sphere complex. Using the Blue-Moon sampling method^41^, we examined the free energetics of the dissolution process by computing the potential of mean force (PMF) along the reaction coordinate defined by the Ca^2+^ z-coordinate, i.e. height above the surface plane. We find that the free-energy barrier for dissolution is 30 kJ/mol indicating that at 323 K this process will be relatively fast. The change of Helmholtz free energy, Δ*A*, between the two minima is 14 kJ/mol demonstrating that at this temperature there will be an equilibrium between inner and outer sphere Ca^2+^ complexes with a *K*_eq_ = exp(−Δ*A*/*k*_B_*T*) ~ 10^−3^. Note that this complex shows a positive increase in free energy as it moves out into the scCO_2_ layer, as long as the first coordination sphere of Ca^2+^ consists only of water. However, complexes with mixed coordination (H_2_O and CO_2_) may further enhance Ca transport away from the surface^17^,^42^.

Ca^2+^ dissolution results in Ca-vacancies where carbonate formation is expected to be spontaneous, in a process similar to sulfite formation^35^. To verify this, we computed a PMF by moving a CO_2_ from the scCO_2_ liquid across the water layer towards the Ca vacancy to estimate the free energy of carbonate formation. As can be seen in Fig. 4b, this process is effectively barrier-less and exergonic by 17 kJ/mol, implying that carbonation can readily occur and is limited only by diffusion and the available population of Ca-vacancy sites. This emphasizes the paramount importance of a stable water monolayer in establishing the nucleation sites for carbonate formation.

### Water adsorption/desorption to/from Anorthite

**Figure 5:**
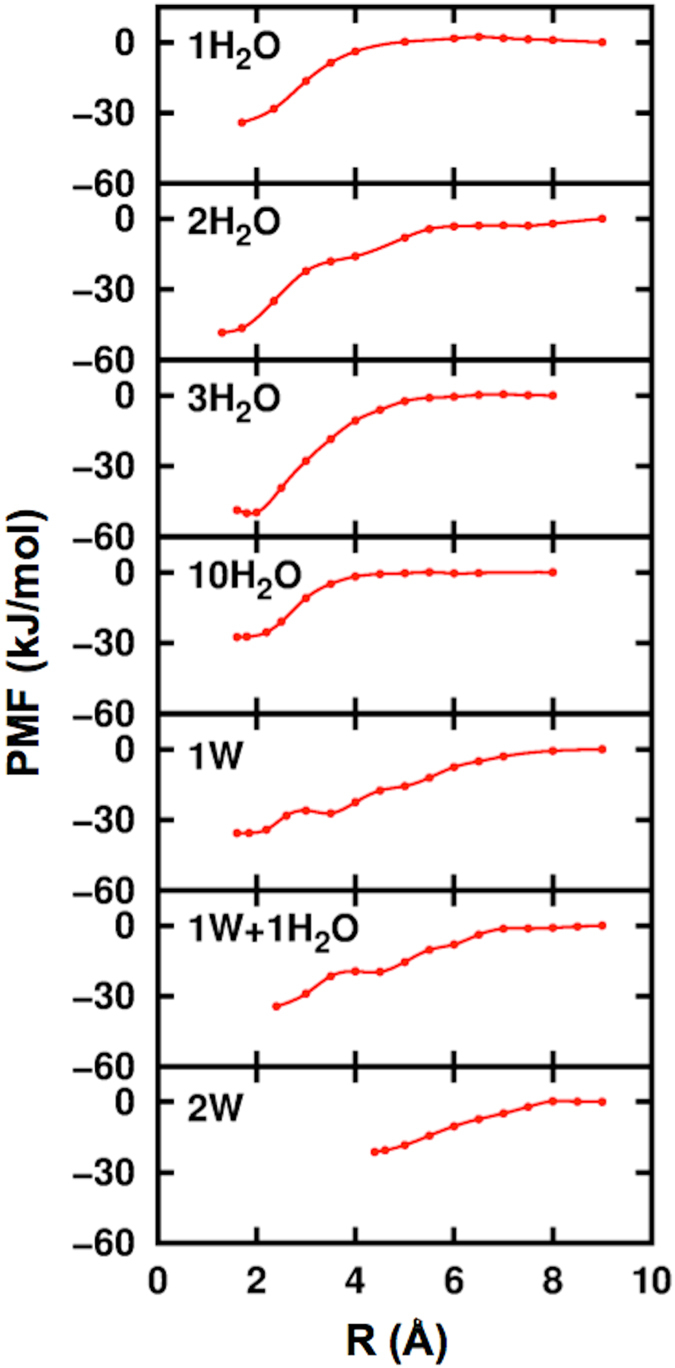
PMF of water layer formation. Computed Potential of Mean Force (PMF) obtained for bringing a water molecule from scCO_2_ phase towards the anorthite surface.

**Figure 6:**
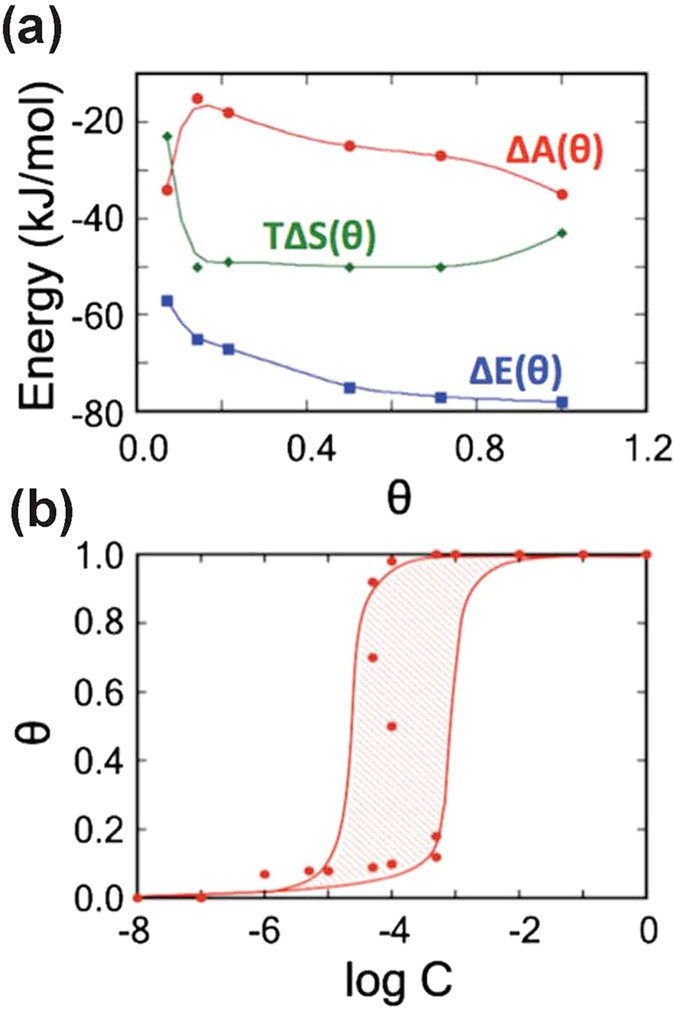
Energetics and Isotherm. (**a**) De-convoluted energetics of water adsorption as a function of surface coverage θ. (**b**) Adsorption isotherm as a function of water concentration *C*.

We now will focus on extracting information about water transport to, from and across these interfaces. This phenomenon is particularly relevant to reactivity under geologic sequestration conditions, as water-saturated scCO_2_ can lead to unexpected events, such as steel corrosion in water-lean scCO_2_^33^,^34^ or preferential sulfite formation in carbonate minerals^35^. We computed the PMF for moving a single H_2_O from the middle of the liquid slab through the box, such that its immediate environment changes from being solvated in the scCO_2_ to being bound on the Ca-rich surface^41^. This process was repeated for the following systems: 1H_2_O/scCO_2_, 2H_2_O/scCO_2_, 3H_2_O/scCO_2_, 10H_2_O/scCO_2_, 14H_2_O/scCO_2_ (1W), 15H_2_O/scCO_2_ (1W+1H_2_O), and 28H_2_O/scCO_2_ (2W). Supplementary Section S4 provides details on the PMF convergence. The associated changes in the Helmholtz free energy, Δ*A*, are given in Fig. 5, and the computed coverage dependent adsorption free energy, Δ*A*(*θ*), is presented in Fig. 6a.

The free energy landscape for the 1H_2_O/scCO_2_ system is essentially flat, up until H_2_O is *ca* 5 Å away from the anorthite surface, see Fig. 5 and Supplementary Fig. 6. This distance corresponds to the water approaching the periphery of the high density CO_2_ layer. The free energy decreases monotonically up to 1.7 Å above the surface where the water is stabilized by forming both Ca-O_w_ and hydrogen bonds with the O atoms of the mineral surface, Δ*A *= −34 kJ/mol. The simulations with 2 and 3 H_2_O molecules were started with a pre-formed water dimer and trimer in the scCO_2_ liquid, see panel 2H_2_O in Supplementary Fig. 6. Because the constrain is imposed on only one of the waters, the density and free energy profiles are very similar to those for 1 H_2_O, with the other H_2_O molecule(s) moving freely. Adsorption occurs step-wise with the H_2_O dimer/trimer breaking apart along the way and with the constrained water molecule adsorbing first. Subsequently, in both the dimer and trimer simulations, the remaining molecules gravitate towards the surface adsorbed H_2_O: once the additional waters become engulfed in the scCO_2_ layer, they adsorb to the surface adjacent to the constrained H_2_O molecule without any additional constraints. These simulations imply that water film growth begins with a single water molecule as a nucleation site followed by the addition of subsequent molecules, to form an island.

To quantify this observation, we focus on the Δ*A(θ)* of the reactions:


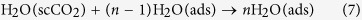


The resulting free energy Δ*A*(*θ*), as a function of coverage *θ* (*θ* = n/14), is plotted in Fig. 5a. We note that in order to directly compare the Δ*A*(*θ*) values obtained from the PMF for the *n *= 2 and 3 simulations (which are relative to solvated clusters) with those of the *n *= 1, 7, 10 and 14 (which are relative to solvated monomers) we adjust the free energy of the cluster in the solvated phase to be relative to *n* solvated water monomers at infinite dilution, see Supplementary Section S6 for details. In accord with our above conclusion regarding H_2_O-H_2_O interactions, the computed Δ*A*(*θ*) values grow steadily from *n *= 2–14 by about −20 kJ/mol. Note that this value is on the order of the molecule-molecule interaction energy (*BE*3 = −15 kJ/mol) reported in Table 1.

We proceed by de-convoluting Δ*A*(*θ*) = Δ*E*(*θ*) − *T*Δ*S*(*θ*) into its entropic (*T*Δ*S*(*θ*)) and energetic (Δ*E*(*θ*)) components in order to understand the role played by the H_2_O-H_2_O interactions in driving film formation. To assess Δ*E*(*θ*), we consider that adsorption of water at the anorthite surface involves replacing adsorbed CO_2_ molecules with adsorbing waters at a ratio of 9CO_2_/14H_2_O based on the full coverage of the of CO_2_ and H_2_O monolayers:


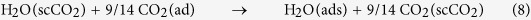


This process can be subdivided into the sum of two half reactions:


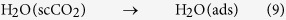



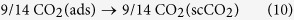


The energy Δ*E*(*θ*) of (9) and (10) can be easily estimated based on arguments similar to those discussed earlier. As shown in the thermodynamic cycle of Fig. 1, for H_2_O, the energy of (9) can be decomposed utilizing the binding energies of Table 1, while introducing an intermediate state (H_2_O(g)) and the solvation energy of water in scCO_2_ (estimated to be −20 kJ/mol^39^). Neglecting the molecule-molecule interaction term (*BE*3) and using only the average surface-molecule adsorption energy (*BE*2) we obtain:


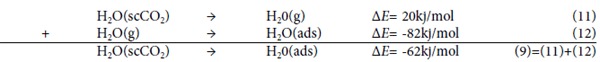


In an analogous manner, the energy for (10) is found to be Δ*E *= 6 or 4 kJ/mol, with or without CO_2_-CO_2_ interactions. As such, the energy for (8) can be estimated to be approximately −57 kJ/mol neglecting H_2_O-H_2_O interactions. To include the latter term, we note that an H-bond between waters in the 1W system was estimated to have an average energy of 15 kJ/mol and the number of hydrogen bonds per bound H_2_O molecule increases steadily from 0.5–1.4 between *n *= 2–14 as estimated from the number of H-bonds/water obtained from the AIMD simulations. Assuming an average stabilization energy directly proportional to the number of H-bonds, Δ*E*(*θ*) smoothly evolves from −57 kJ/mol (*n *= 1) to −78 kJ/mol (*n *= 14) as shown in Fig. 6a.

The relationship Δ*E*(*θ*) **−** Δ*A*(*θ*) = *T*Δ*S*(*θ*) allows us to estimate the entropy change during adsorption, which agree fairly well with values obtained using quasiharmonic approximation^43^, see Supplementary section S7 for detailed method and results. In all cases, the entropy of adsorption (Fig. 6a) is negative due to a loss of the degrees of freedom of the scCO_2_-solvated H_2_O upon adsorption. The function *T*Δ*S*(*θ*) becomes more negative by −27 kJ/mol between the adsorption of a monomer and a dimer, then remains relatively flat as the cluster of adsorbed waters increases in size up to a full monolayer. This occurs because addition of water molecules to a surface-bound cluster requires an increase in entropy of adsorption due to the restriction of the number and locality of binding sites. This factor is roughly unchanged for *n *= 2–14 and hence *T*Δ*S*(*θ*) is relatively constant as the layer forms. As such, Δ*A*(*θ*) decreases roughly at the same rate as Δ*E*(*θ*), and is proportional to the increase in the H-bonding. Our simulations indicate that the barrier for nucleation of a 1W water layer, via an island growth mechanism, arises due to the entropic considerations and has the strongest influence at *n *= 2–3 where the number of H-bonds are relatively few.

Finally, our results also suggest that water layer formation will be kinetically hindered due to the lack of a strong thermodynamic driving force at low *θ*. To assess this quantitatively, we have computed an adsorption isotherm^44^ employing the *θ* -dependent equilibrium constants, *K*(*θ*) = exp(−Δ*A*(*θ*)/*k*_B_*T*), see Fig. 6b. From this isotherm, we solve the equation to estimate the expected water coverage as a function of water concentration. We find that water concentrations below 10^−5^%mol result in very low surface coverage (*θ *< 0.1), while for water concentrations above 10^−3%^mol, a full coverage water monolayer is expected. In the intermediate concentration range of 10^−5^**−**10^−3^, consistent with the average 10^−4^%mol concentration of water in wet scCO_2_ at *P *= 90 bar, *T *= 323 K^45^, the non-monotonic behavior of Δ*A*(*θ*) leads to multiple solutions to the equation where both a high and low coverage regions are stable. This is interpreted as the likely occurrence of an equilibrium distribution of high and low coverage patches on the surface. In the context of Ca^2+^ extraction and carbonate formation, this implies that the partial water layer formation will impact the population of Ca^2+^ vacancies and thus be a major kinetic bottleneck for carbonate formation.

## Discussions

We have demonstrated that in the case of a prototypical mineral surface, here anorthite, in contact to water-saturated scCO_2_, a liquid, boundary layer of water will form and facilitate the extraction of Ca^2+^ cations in the form of stable, hydrated, outer-sphere complexes. This process creates cation vacancies at the top-most layers, which can serve as nucleation sites for carbonate formation limited only by the water layer formation and CO_2_ diffusion. Water film formation was found to be energetically driven by both Ca^2+^-H_2_O and H_2_O-H_2_O interactions, but entropic contributions largely override the stabilizing factors. The entropic component arises from H_2_O adsorption on the surface as well as the availability of binding sites. Our results highlight that the existence of a boundary water layer, significantly different in composition to the bulk liquid phase, can occur at even relatively low concentrations, as in the case of water-bearing scCO_2_. In the present case, it also points to a novel mechanism for carbonation, distinct from the traditional view that carbonation must begin with CO_2_ dissolution in the water phase. We have shown that a molecular level understanding of enthalpic and entropic contributions is now achievable through a combination of simple and intuitive chemical arguments and large-scale AIMD simulations. With this capability, we can now access an unprecedented level of qualitative and quantitative molecular scale understanding of structure, dynamics and reactivity at a chemically complex solid-liquid interface which may be difficult to obtain by direct experimental observation alone.

## Methods

### DFT parameters

Density-functional-theory(DFT) based AIMD simulations were carried out with periodic boundary conditions (3D PBC) with the exchange correlation functional of Perdew, Burke and Ernzerhoff (PBE)^46^ as implemented in the CP2K package^25^. Dispersion corrections were accounted for by Grimme’s third generation corrections DFT-D3^47^ that have been evaluated extensively in the literature for both scCO_2_ and H_2_O and shown to adequately represent weak interactions in these systems^16^,^17^,^38^,^48^,^49^,^50^. The core electrons were described by the norm-conserving pseudopotentials^51^, while the valence wavefunctions were expanded in terms of double-zeta quality basis sets optimized for condensed systems to minimize linear dependencies and superposition errors^52^. An additional auxiliary plane wave basis set with a 350 Ry cutoff was used for the calculation of the electrostatic terms. We used a time-step of 1.0 fs and the Nosé-Hoover thermostat for NVT ensemble calculations at 323 K.

### Computational models

Anorthite, with the chemical composition of CaAl_2_Si_2_O_8_, is the calcium end-member of the plagioclase feldspars. A coupled substitution of two Al^3+^ for two of Si^4+^, allows the incorporation of one Ca^2+^ and overall neutrality^53^. The fully optimized cell parameters at P=90 bar, *a *= 8.231 Å, *b *= 12.98 Å, *c *= 14.15 Å, *α *= 93.9°, *β *= 115.4°, γ = 91.3°, are less than 1% different than the crystallographic parameters, *a*_0_ = 8.1860 Å, *b*_0 _= 12.876 Å, *c*_0_ = 14.1820 Å, *α *= 93.3°, *β *= 115.8°, γ = 91.12°. Using the optimized parameters, an oxygen-terminated (001) surface model was generated, shown in Supplementary Fig. 1. A 2 × 1 × 1 slab model of the (001) surface (one of the most frequently occurring surfaces in this class of minerals^23^,^54^) with 18.3 Å interlayer space was created, including the top-most layer of Ca cations (Ca-rich surface) in order to study Ca-speciation and mobilization. The bottom side of the slab is devoid of Ca cations (Ca-deficient surface) in accordance with known cation vacancies as reported in the literature^23^. Finally, 8 hydrogens were added to terminal oxygens of the upper surface of the slab for charge compensation.

The mineral-liquid interface was modeled with 2 different simulations of the anorthite slab with 32 CO_2_ or 84 H_2_O molecules in the interlayer space, determined by optimization of cell parameters at 90 bar. MD simulations within the NVT statistical ensemble were performed at 323 K, resulting in a pronounced peak near the surface in the density profile plot indicating formation of strong first H_2_O/CO_2_ layer near the interface. Based on this analysis, the first layer of H_2_O/CO_2_ was determined to contain 14H_2_O/9CO_2_ molecules respectively and was used to conduct simulations for stand-alone monolayer properties.

### AIMD simulations

Simulations with 1 H_2_O/32 CO_2_, 2 H_2_O/31 CO_2_ and 3 H_2_O/30 CO_2_ molecules were performed to simulate very low hydration states. In addition, simulations with 10/28, 14/28, 15/28 and 28/21 H_2_O/CO_2_ molecules were performed to simulate the formation of 1 (1W) and 2 (2W) monolayers. Because 14 was found to be the least number of H_2_O required to form a full monolayer, the simulation with 15 H_2_O was performed to provide estimates of H_2_O binding onto the first stable ad-layer. In all cases, full cell optimizations of the composite systems were performed at 90 bar. Supplementary Table 1 summarizes the optimized simulation cell parameters and simulation times for each system. Estimates of the diffusion coefficients and densities in the liquid slab of each simulation are summarized in Supplementary Table 2. Overall, it was found that the scCO_2_ layer in our model adequately reproduces the structure, density and dynamics of an scCO_2_ liquid phase at ~90 bar and *T *= 323 K.

We have employed the Blue-Moon ensemble method^41^ to calculate the potential of mean force (PMF) required to move a H_2_O molecule from the middle of the liquid region towards the Ca-rich face of the anorthite (001) surface. This method is broadly used to compute the free energy profile along a reaction coordinate direction, which is not likely explored with MD simulation due to potentially high barriers. For the initial and final points, we also performed unconstrained MD simulations to locate the thermodynamic minima. The average forces subject to geometric constraints were calculated as ensemble-averages, and converted to a PMF based on the Blue-Moon ensemble method^41^: the *z*-distance between the surface and the water Oxygen atoms was chosen as the geometric constraint, and all other parameters were allowed to vary. This approach allowed the H_2_O molecule to move laterally over the mineral surface through the liquid boundary layer. Integrating the average force along the reaction coordinate generated the free energy profile. Increments in *dz* were chosen to be 0.2–0.5 Å apart depending on the steepness of the PMF to better estimate the free-energetics via thermodynamic integration, which resulted in a total of 11–18 simulations per PMF. The force on each point in the PMF was converged to within ±0.0004 a.u. See Supplementary section S4 for details.

Each AIMD trajectory, both constrained and unconstrained, was equilibrated for approximately 5–10 ps and data was collected for analysis from at least 10 ps of well-equilibrated trajectory. The sum total of the overall compiled AIMD trajectories accounts for approximately 2.0 ns of total simulation time (system-specific simulation times are recorded in Supplementary Table 1). This data set represents over 10^6^ DFT configurations for systems of total size of ~500 atoms.

## Additional Information

**How to cite this article**: Lee, M.-S. *et al.* Structure, dynamics and stability of water/scCO_2_/mineral interfaces from *ab initio* molecular dynamics simulations. *Sci. Rep.* **5**, 14857; doi: 10.1038/srep14857 (2015).

## Supplementary Material

Supplementary Information

## Figures and Tables

**Table 1:** Binding energies of H_2_O and CO_2_ at the Ca-rich anorthite (001) surface.

System	BE_g_	BE1	BE2	BE3	CE^a^	BE4
H_2_O	−103	−97	−82	−15	−57	−40
CO_2_	−34	−17	−13	−4	−7	−10

See text for definitions. Energies are in kJ/mol.

^a^Experimental values of heat of vaporization of H_2_O and CO_2_ at 221 K are 47.048 and 15.049 kJ/mol, respectively.
