# Supplementary material for: Structure, dynamics and stability of water/scCO2/mineral interfaces from ab initio molecular dynamics simulations
Source: Sci Rep. 2015 Oct 12;5:14857. doi: 10.1038/srep14857 (PMC4600984; doi:10.1038/srep14857)
Supplement: Supplementary Information [file srep14857-s1.pdf]

Supporting Information

Structure, dynamics and stability of water/scCO$_2$/mineral interfaces from ab initio molecular dynamics simulations

Mal-Soon Lee[1], B. Peter McGrail[2], Roger Rousseau[1], Vassiliki-Alexandra Glezakou[1*]*

[1]Fundamental and Computational Sciences Directorate, [2]Energy and Environment Directorate, Pacific Northwest National Laboratory, Richland, WA 99352

S1. Simulation cell and structure of H_2O and CO_2 monolayers.

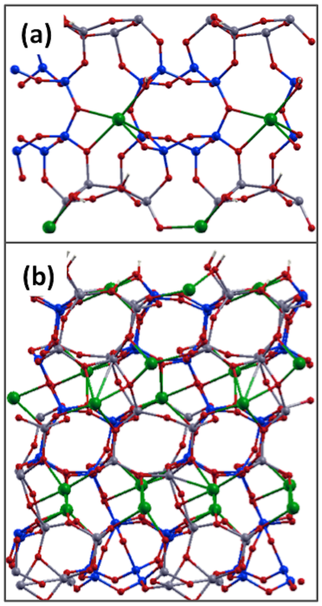

Figure 1. Structure of the Anorthite (001) slab used in this study: Atoms are represented by balls with color: Blue (Si), Grey (Al), Red (O), White (H) Green (Ca).

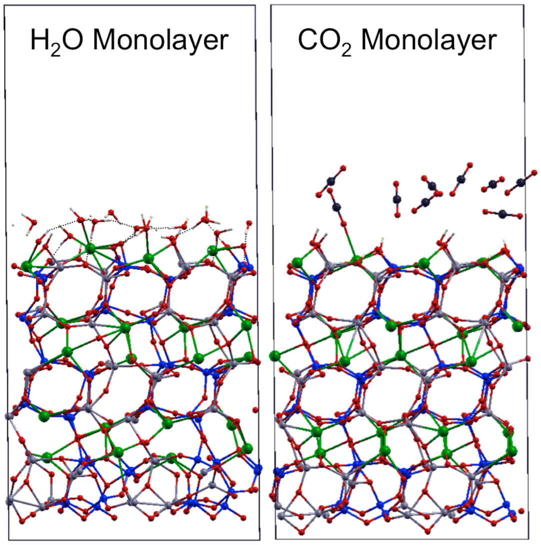

Figure 2. Structure of full coverage water and $scCO_2$ adlayer on the Ca-rich Anorthite (001) adlayer: H (white), C (black), O (red), Si (blue), Al (gray), and Ca (green).

S2. Summary of simulation cell parameters and validation of liquid state of water and $scCO_2$ layers from AIMD simulations.

Table 1. Optimized cell parameters and simulation time.

System	Cell parameters	Interlayer spacing (Å)	Simulation time (ps)
**$32CO_2$**	a=16.42 Å, b=13.10 Å, c=34.64 Å<br>α=90.1°, β=90.5°, γ=91.6°	18.3	15
**$14H_2O$**	a=16.43 Å, b=13.07 Å, c=34.97 Å<br>α=90.0°, β=90.5°, γ=91.4°	18.3	15
**$1H_2O/32CO_2$**	a=16.42 Å, b=13.10 Å, c=34.64 Å<br>α=90.1°, β=90.5°, γ=91.6°	18.3	225*
**$2H_2O/31CO_2$**	a=16.42 Å, b=13.10 Å, c=34.64 Å<br>α=90.1°, β=90.5°, γ=91.6°	18.3	224*
**$3H_2O/30CO_2$**	a=16.42 Å, b=13.10 Å, c=34.64 Å<br>α=90.1°, β=90.5°, γ=91.6°	18.3	241*
**$10H_2O/28CO_2$**	a=16.41 Å, b=13.07 Å, c=33.25 Å<br>α=89.9°, β=90.6°, γ=91.5°	17.0	294*
**$14H_2O/28CO_2$**	a=16.43 Å, b=13.07 Å, c=34.97 Å<br>α=90.0°, β=90.5°, γ=91.4°	18.3	428*,†
**$15H_2O/28CO_2$**	a=16.43 Å, b=13.07 Å, c=34.97 Å<br>α=90.0°, β=90.5°, γ=91.4°	18.3	246*
**$28H_2O/21CO_2$**	a=16.43 Å, b=13.07 Å, c=35.09 Å<br>α=90.1°, β=90.6°, γ=91.3°	18.5	234*
**$42H_2O/20CO_2$**	a=16.42 Å, b=13.07 Å, c=36.12 Å<br>α=90.1°, β=90.5°, γ=91.2°	19.2	28
**$84H_2O$**	a=16.39 Å, b=13.07 Å, c=36.07 Å<br>α=90.2°, β=90.5°, γ=91.1°	19.3	20

* Total time for both constraint and unconstraint MD simulations.

† Simulations for Ca positions and CO_3 formation are also included.

Table 2. Summary of simulation cell parameters and validation of liquid state of water and $scCO_2$ layers from AIMD simulations.

Trajectory	Diffusion Coefficient[a] ($10^{-4}cm^2/s$)	Density[b] (g/cm^3)
**$scCO_2$**	1st layer: D_{xy}=0.3, D_z=0.2<br>Liquid: D_{xy}=1.4, D_z=0.4	1st layer: 1.2<br>Liquid: 0.40
**Water**	1st layer: D_{xy}=0.02, D_z=0.01<br>Liquid: D_{xy}=1.0, D_z=0.2	1st layer: 1.9<br>Liquid: 1.02
**1W**	1st layer: D_{xy}=0.03, D_z=0.01	1st layer: 2.1
**1W/$scCO_2$**	1st layer: D_{xy}=0.01, D_z=0.01<br>CO_2 Layer: D_{xy}=1.1, D_z=0.9<br>CO_2 Liquid: D_{xy}=1.0, D_z=0.3	1st layer: 1.9<br>CO_2 Layer: 0.84<br>CO_2 Liquid: 0.49
**2W/$scCO_2$**	1st layer: D_{xy}=0.02, D_z=0.01<br>CO_2 Liquid: D_{xy}=1.0, D_z=0.3	1st layer: 1.8<br>CO_2 Liquid: 0.29
**3W/$scCO_2$**	1st layer: D_{xy}=0.02, D_z=0.01<br>CO_2 Liquid: D_{xy}=1.0, D_z=0.3	1st layer: 1.9<br>CO_2 Liquid: 0.39

[a]Diffusion Coefficient of $scCO_2$ $5.2x10^{-4}cm^2/s$ (P=101bar,T=323K)[1] , H_2O $0.35x10^{-4}cm^2/s$ (P=101 bar, T=3218)[2]. [b]Density of $scCO_2$, $0.4g/cm^3$, H_2O $1.02g/cm^3$ (T=323K, P=100bar).

The computed values of the diffusion coefficient (D) and Density for water and CO_2 are given in **Supplementary Table 2**. The D values were extracted from the slope of the mean square displacement correlation function and decomposed into components that are parallel, D_{xy}, and perpendicular, D_z, to the surface, see **Supplementary Figure 3** for the 1W/$scCO_2$ case. In both cases the density and diffusion are satisfactorily comparable to values measured experimentally.

---

[1] P. Etesse, J. A. Zega, and R. Kobayashi, J. Chem. Phys. 97, 2022 (1992).
[2] Harris, Kenneth R., and Lawrence A. Woolf. *J. Chem. Soc., Faraday Transactions 1:* 76 (1980): 377-385.

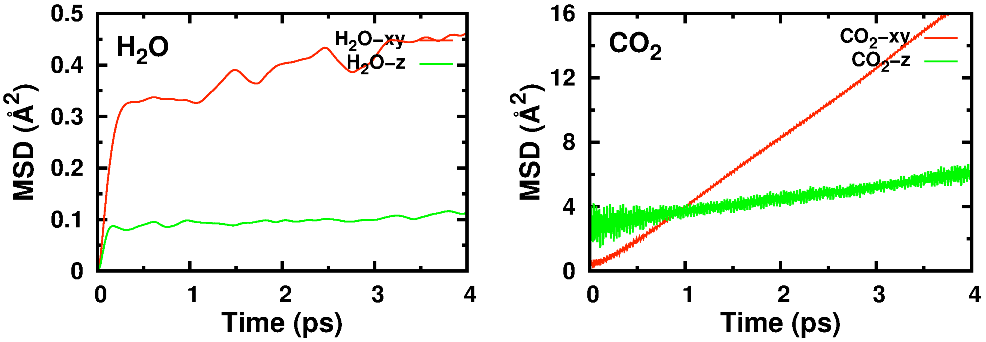

Figure 3. MSD for AIMD trajectory of H_2O (left) and $scCO_2$ (right) for $1W/scCO_2$ layer.

S3. Atomic density profiles following Ca dissolution.

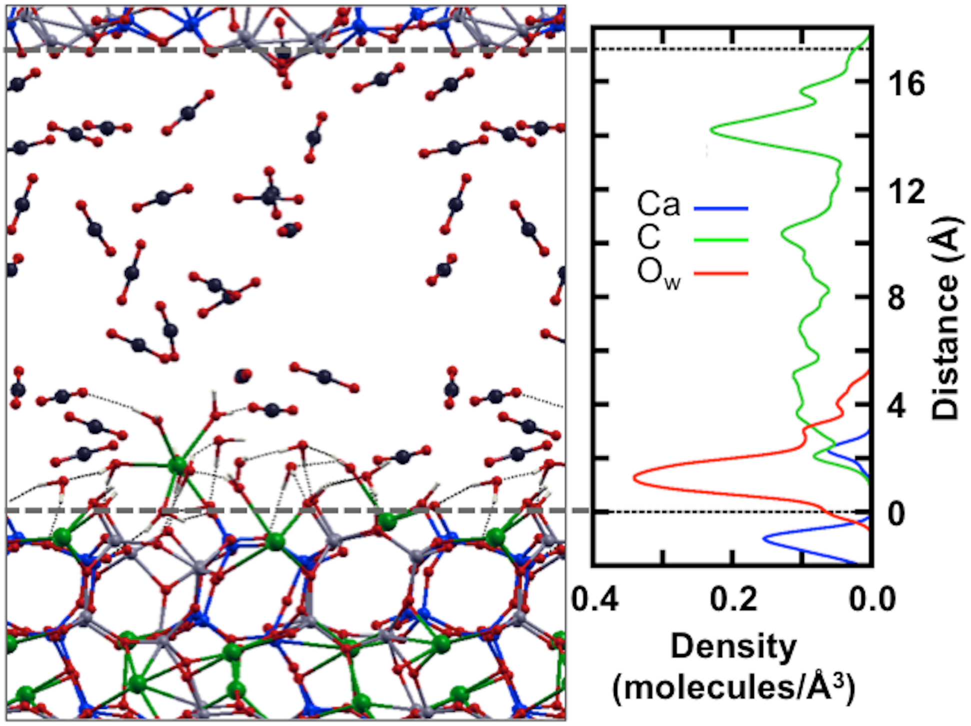

Figure 4. (a) Snapshot of liquid phases from AIMD runs at 323 K with Ca^{2+} ion solvated in 1W layer, Hydrogen (white), Carbon (dark grey), Oxygen (red), Silicon (blue), Aluminum (silver), Calcium (green). (b) Molecular density projected along the direction of the surface normal, Red (water), Green (CO_2), Blue (Ca).

S4. Convergence of PMF.

To calculate potential mean force (PMF), we employed constrained MD based on the Blue-Moon sampling method: at each point in the PMF (11-18 points) a short (~10 ps) simulation was performed. In **Supplementary Figure 5,** we plot the expectation value of our constrained force, <F>, as a function of our reaction distance, z coordinate of O_w, for selected PMFs. Error bars on <F> are estimated to be at most ±0.0004 a.u. As a rough estimate, an average bin with width of 0.4 Å gives an approximate error for each point in our integration of *ca* ±2 kJ/mol. A more detailed error analysis in the numerical integration of the force functions suggest an overall convergence of ±2kJ/mol for the ΔA values reported in the text.

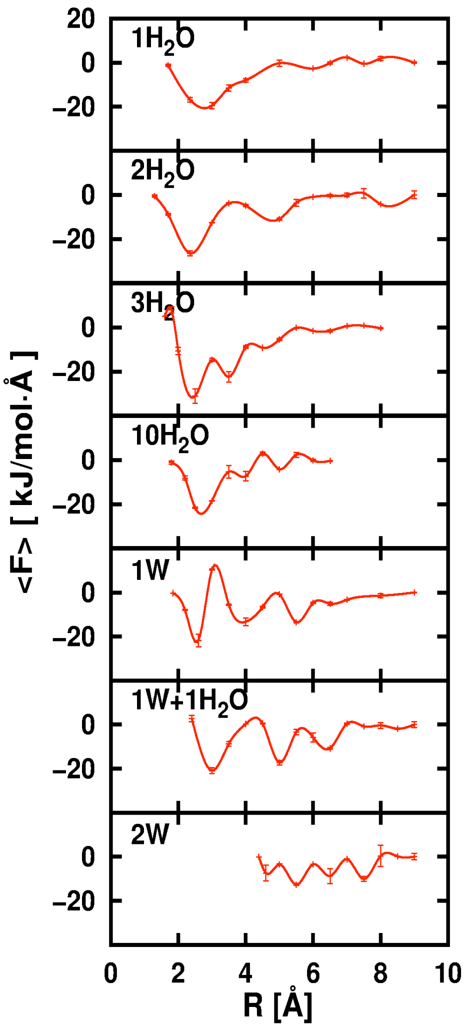

Figure 5. Expectation value of constrained force <F> in kJ/mol·Å. as a function of reaction distance. Error bars are estimated via a plateau analysis[1].

S5. Density profiles from selected PMFs

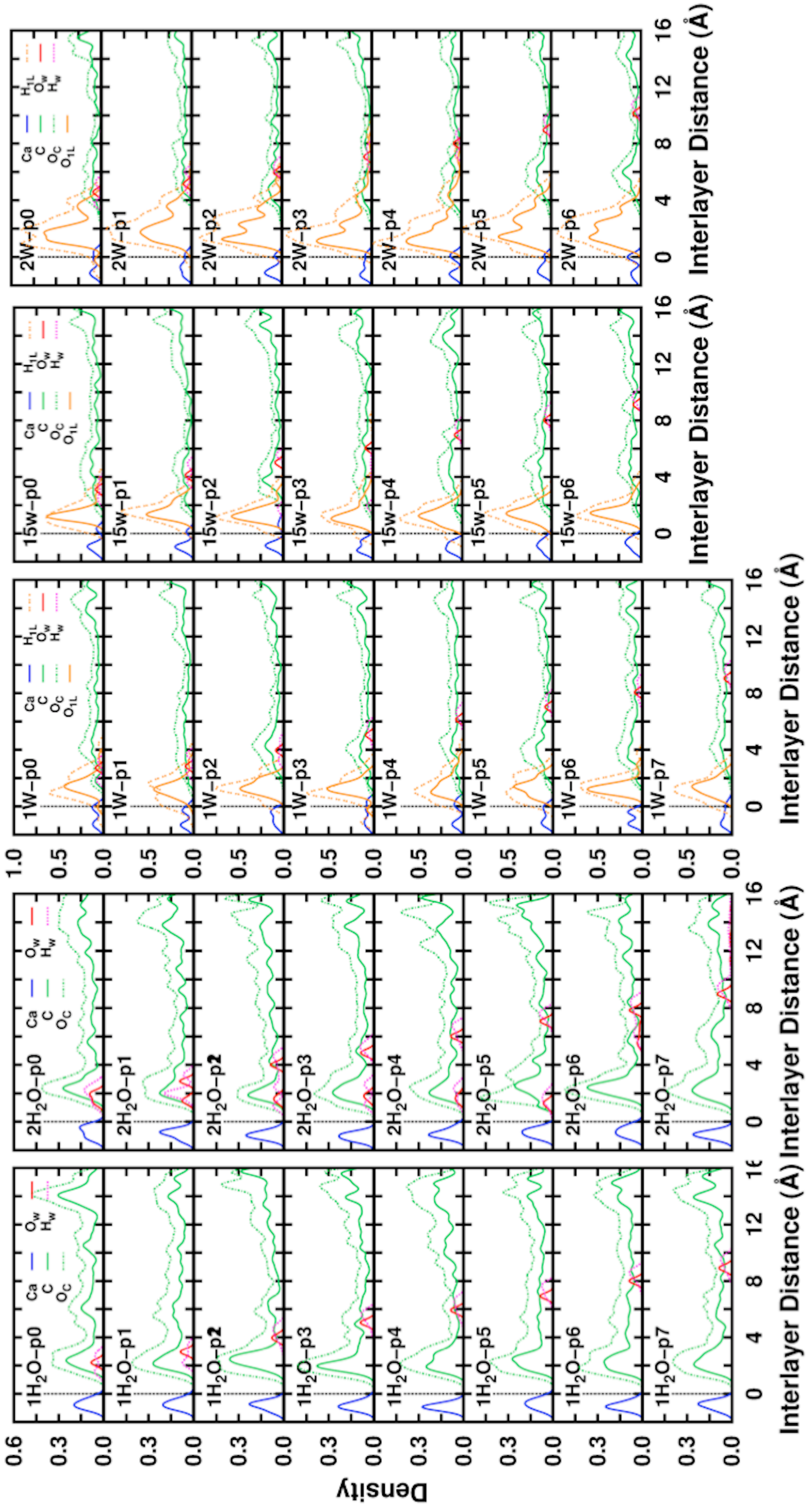

Figure 6. Molecular Density profiles for points along selected PMFs showing how molecular layers are perturbed by the constrained H_2O molecules during the MD. Color Scheme same as Figure 7 in main text.

S6. Converting dimer and timer ΔA to correct reference state.

As discussed in the text the dimer (*n*=2) and trimer (*n*=3) simulations are performed with regards to a reference which is different from that of the *n*=1,7,18 and 14 simulations. We can however adjust the resulting ΔA values by the following arguments:

For the dimer we performed the simulation:

$(H_2O)_2(scCO_2) \rightarrow 2H_2O(ads)$ ΔA=-49 kJ/mol (RS2-1)

To make this consistent with *n*=7-14 we need to compute:

$H_2O(ads)+H_2O(scCO_2) \rightarrow 2H_2O(ads)$ (RS2-2)

The dimer can be converted to a reference state of two monomers by the following chemical reactions:

$2H_2O(scCO_2) \rightarrow (H_2O)_2(scCO_2)$ ΔA=-1 kJ/mol[3] (RS2-3)

The adsorption of 1 monomer relative to a dimer can be expresses as:

$(H_2O)_2(scCO_2) \rightarrow H_2O(ads)+H_2O(scCO_2)$ ΔA=-33 kJ/mol (RS2-4)

The free energy of (RS2-5) can be obtained by the following relationship (RS2-3)+(RS2-4) = (RS2-5):

$2H_2O(scCO_2) \rightarrow H_2O(ads)+H_2O(scCO_2)$ ΔA=-34 kJ/mol (RS2-5)

which is the reaction for the monomer obtained in the text.

Now subtracting reaction RS2-4 from RS2-1 provides reaction RS2-2. Hence:

$H_2O(ads)+H_2O(scCO_2) \rightarrow 2H_2O(ads)$ ΔA=-15 kJ/mol (RS2-2)

Similarly for the trimer. We have computed:

$(H_2O)_3(scCO_2) \rightarrow 3H_2O(ads)$ ΔA=-50 kJ/mol (RS3-1)

We require:

$2H_2O(ads)+H_2O(scCO_2) \rightarrow 3H_2O(ads)$ (RS3-2)

To achieve this, we note that:

$3H_2O(scCO_2) \rightarrow (H_2O)_3(scCO_2)$ ΔA=-17 kJ/mol[1] (RS3-3)

$(H_2O)_3(scCO_2) \rightarrow H_2O(ads)+2H_2O(scCO_2)$ ΔA=-17 kJ/mol (RS3-4)

The energies of (RS3-4) comes from the use of the monomer reaction (-34 kJ/mol) and reaction (RS3-3). We can rewrite reaction (RS2-2) as reaction (RS3-5):

---

[3] Glezakou, V. A.; Rousseau, R.; Dang, L. X.; McGrail, B. P. *Physical Chemistry Chemical Physics* **2010**, *12*, 8759.

$H_2O(ads)+2H_2O(scCO_2) \rightarrow 2H_2O(ads)+H_2O(scCO_2)$ ΔA=-15kJ/mol (RS3-5)

Summing (RS3-3) and (RS3)-5 provides:

$(H_2O)_3(scCO_2) \rightarrow 2H_2O(ads)+H_2O(scCO_2)$ ΔA=-32kJ/mol (RS3-6)

Finally subtracting (RS3-6) from (RS3-1) provides:

$2H_2O(ads)+H_2O(scCO_2) \rightarrow 3H_2O(ads)$ ΔA=-18kJ/mol (RS3-2)

S7. Entropy change using quasiharmonic approximation

To verify the entropy changes estimated from the Blue-Moon ensemble calculations reported in the text, we calculated entropies using a quasi-harmonic approximation where translational, rotational, and vibrational entropies are calculated from the principal root-mean-square fluctuations for the center of mass of H_2O molecule, the principal moments of inertia of a H_2O molecule, and vibrational density of states (VDOS), respectively. Detailed description of this approach can be found in the reference 4[4]. For the translational and rotational entropies, we used unconstrained MD simulation conducted with a configuration with one H_2O molecule in the middle of the interlayer, while the vibrational entropy is calculated using constrained MD simulations conducted at two end positions (with H_2O in the middle of the $scCO_2$ phase and the other with H_2O at the surface). We have chosen two systems with 10 H_2O and 14 H_2O representing significant changes in entropy. The Table below shows the values of TΔS obtained using the two independent methods.

System	Blue-Moon method (kJ/mol)	Quasi-harmonic analysis (kJ/mol)
$10H_2O$	-49±3	-60±9
$14H_2O$	-43±2	-52±8

The entropic contribution from translation and rotation is ~40 kJ/mol for both systems. We should point out that convergence of the VDOS calculation is much slower than that for the force calculation. Error analysis of entropy from VDOS shows a larger error (~15 %) than that with Blue-Moon method (~5 %). Thus results from two methods agree fairly well, giving confidence to our discussion about free energetics.

[4] Carlsson, J. and Åqvist, J. *The Journal of Physical Chemistry B* **2005**, 109, 6448-6456.

S8. Cartesian coordinates and cell parameters of structures employed in binding energy analysis.

A. Anorthite slab + 14 H_2O

CRYSTAL
PRIMVEC

16.4340235543	0.0000000000	0.0000000000
-0.3203164176	13.0680953535	0.0000000000
-0.2856096309	0.0038537095	34.9691087411

PRIMCOORD

358 1

Si	6.8850926111	10.6133596982	1.6770133577
Si	15.0910314798	10.6113212861	1.6732846128
Si	4.2289296540	9.8067613651	1.9096187243
Si	12.4545188432	9.8092317653	1.8758843031
Si	6.6438712353	5.5665535246	2.2467199291
Si	14.8769109363	5.5623647355	2.2732292394
Si	12.2467109593	6.8691059977	2.3174123334
Si	3.9994344309	6.8729009176	2.3138754535
Si	0.0880322662	9.8837008947	4.2288309439
Si	8.3354421876	9.8973983655	4.2096410661
Si	8.1630810463	6.7678238120	4.4034399060
Si	-0.0551632076	6.7605124877	4.4543055033
Si	5.3588496266	10.7080718558	4.8643211528
Si	13.5609925195	10.7060111497	4.8576054332
Si	5.1049458112	5.8408238223	4.8777615969
Si	13.3313104521	5.8460610733	4.9093824197
Si	12.2290299909	10.1387781643	7.6567021885
Si	4.0318725598	10.1383950142	7.6629106518
Si	4.1459620368	5.5178351267	7.7709169928
Si	12.3466293699	5.5395869387	7.7865379003
Si	9.3747830075	9.3032920829	8.4269931772
Si	1.1913087224	9.3104627023	8.4488018587
Si	1.1659485691	6.2473811760	8.6171763139
Si	9.3509607811	6.2414767206	8.6130782002
Si	13.5593191630	9.3638265724	10.3452933629
Si	5.3548298709	9.3605287670	10.3497938552
Si	5.2524093489	6.3152876758	10.5861345140
Si	13.4745524107	6.3221694607	10.6153515252
Si	10.5789896146	10.1198520845	11.2153012343
Si	2.3897592362	10.1227709397	11.2525666581
Si	2.4680026404	5.4445644890	11.2666685582
Si	10.6700237820	5.4553388425	11.2579519627
Si	9.2812823226	9.6023380938	14.0057584228
Si	1.0605663343	9.6127726192	14.0249527926
Si	1.1483256849	4.9628288362	14.0906964002
Si	9.4001669658	4.9444155450	14.0853339098
Si	6.4599332422	8.7559434567	14.5510025590
Si	14.6663783325	8.7805282182	14.5941146299
Si	6.3927694156	5.7116280770	14.8453654218
Si	14.6103988873	5.7497172271	14.8168896091
Si	10.4444617787	8.8099273186	16.7023309894
Si	2.2324137480	8.8430109864	16.7605966955

Si	2.2137668712	5.8462331458	16.9088610185
Si	10.5214453233	5.8174156061	16.8632991772
Si	7.4042030039	9.4070690379	17.4819717333
Si	15.6355389167	9.4023741758	17.5521794199
Si	15.8013714062	5.0173378980	17.5945382325
Si	7.6300118682	4.9883511689	17.5976201616
Al	8.4648782759	3.2771297128	1.5829864263
Al	0.2756383911	3.2818928293	1.5650381895
Al	2.9322290340	12.4985940180	1.6087722071
Al	11.1175181803	12.4925221123	1.6016238861
Al	10.8605789873	4.1160992806	1.7493815210
Al	2.6675430574	4.1107418207	1.7491103973
Al	8.3542966216	0.3005735115	2.2045466298
Al	0.1568864359	0.3100363416	2.1897964874
Al	13.0750310181	3.3693304829	3.6784418168
Al	4.8526370203	3.3538260950	3.6862296907
Al	12.7276558008	0.4737027280	4.0600851567
Al	4.5070500491	0.4600857325	4.0770765312
Al	10.1276573254	4.3166248589	4.7041297302
Al	1.9262155567	4.3136739930	4.7013141320
Al	9.4298560208	12.5427766579	4.8954170484
Al	1.1772568173	12.5392867922	4.8755781433
Al	8.5832425537	3.5237873500	7.4913470666
Al	0.3715694003	3.5378979112	7.4840234208
Al	-0.0838584020	12.0089551888	7.8316073872
Al	8.1111681065	12.0059445333	7.8221240342
Al	5.5619699556	2.7703903122	8.1967849108
Al	13.7969311820	2.8052813591	8.2259018350
Al	5.0453756229	12.8370728134	8.4266493987
Al	13.2735657088	12.8471361798	8.4752157872
Al	1.6206618153	2.8234728884	10.3291446512
Al	9.8186536990	2.8236586407	10.3331703296
Al	1.1001084045	12.8532076769	10.8270245922
Al	9.3010686544	12.8512089263	10.8252978204
Al	6.7995012501	3.5490681822	11.1564995338
Al	15.0453158942	3.5648176891	11.2002040568
Al	14.5273427524	12.1268450741	11.3411844308
Al	6.3158729696	12.1273291469	11.2933927649
Al	5.3279321330	2.9799841509	13.9915143876
Al	13.5854722949	2.9656099445	14.0430415264
Al	4.9337381368	11.4818982833	14.1188821672
Al	13.1680336415	11.5218554208	14.2133737689
Al	2.3885635671	2.1638223361	14.6583654739
Al	10.6301726818	2.1715570121	14.6298647495
Al	1.8761584276	12.1983217978	14.9493821681
Al	10.0716128005	12.2077345533	15.1355445578
Al	6.6120783685	2.2201946989	16.8677090749
Al	14.9628569710	2.2148686951	16.9045126398
Al	6.2069137471	12.2537816542	17.0220636188
Al	14.4728207193	12.2680225152	17.1078840738
Al	3.4845819578	3.1500394864	17.6713471430
Al	11.8295368530	3.1696111570	17.6590031598
Al	3.1465199863	11.6252579578	17.7826885495

Al	11.3877334180	11.5601691307	17.8416583472
O	9.7787665930	3.6185146754	0.5212278210
O	1.5945819130	3.6220872238	0.5136024199
O	13.9183121349	9.7276402551	1.0381451971
O	5.7006780559	9.7151153855	1.0951443751
O	11.4232767241	10.8185454975	1.2347575155
O	3.2329929011	10.8263207743	1.2350990007
O	7.1320191750	4.4314528719	1.3126440955
O	15.3768497806	4.4414487772	1.3266610548
O	1.6663814111	-0.1335651597	1.4706891439
O	9.8524548565	-0.1352729082	1.4606168679
O	6.9278103749	12.1203648978	1.3839106515
O	15.1764341772	12.1159907899	1.3819714241
O	15.9226316216	1.8273972838	1.9049434812
O	7.6786310929	1.8286959233	1.9774137968
O	5.4650917686	6.5368021393	1.6763347986
O	13.7096244823	6.5326382302	1.6784555082
O	3.5098109583	8.3636772347	1.9002652271
O	11.7579142063	8.3582147325	1.8973088900
O	11.0803253898	5.8690412767	2.0368322559
O	2.8355108339	5.8695560930	2.0351008588
O	12.3258034715	3.3341878867	2.1830802274
O	4.1368096205	3.3334500333	2.1763935396
O	12.7570308948	0.0671195286	2.4041874035
O	4.5727800330	0.0718532171	2.4134128159
O	-0.3365292231	9.8012298053	2.6305932526
O	7.9178612796	9.8112503099	2.6114639344
O	7.8469191093	6.5386145119	2.8430144921
O	-0.3895765146	6.5495278966	2.8992765750
O	9.6076194750	3.7956596980	3.1261663455
O	1.4171690650	3.7832811865	3.1242991667
O	13.0181062999	10.0961719756	3.4384546137
O	4.7521833071	10.0884436010	3.4827292942
O	14.2340410433	4.8672102489	3.7644675125
O	5.9914978135	4.8682680215	3.7287741160
O	8.3516565385	-0.1449427334	3.9318567006
O	0.1091306504	-0.0852598778	3.9283480583
O	12.5722887248	7.0498883688	4.0049210822
O	4.3220436142	7.0483500912	4.0020133861
O	1.3725249261	10.7459232501	4.5967815340
O	9.6261515257	10.7452316200	4.6024972713
O	8.7378366516	8.3406307153	4.6757985343
O	0.4899852881	8.3409352132	4.7384354677
O	13.5737886825	1.9539007063	4.5237398791
O	5.3197069597	1.9537141615	4.5779945900
O	11.1921454748	0.4324535152	4.8730000090
O	2.9639454897	0.3756850696	4.8680122364
O	5.2069354467	12.2734587463	4.9569477048
O	13.3933180958	12.2706172660	4.9598671483
O	3.8686979476	4.5687857854	4.8037697479
O	12.0973473828	4.5728204061	4.8106402179
O	6.9042266314	10.0978747156	5.0386027146
O	15.1085608560	10.1053306168	5.0879877629

O	1.2755306308	6.0187310481	5.0272153817
O	9.4700306380	6.0174657250	5.0227685051
O	6.7359739574	6.6831170502	5.2538526478
O	14.9641358180	6.6704061232	5.3227498725
O	9.5911433908	3.3433264654	6.0344119648
O	1.3983963081	3.3445024462	6.0382391895
O	12.8485335512	9.7907402939	6.1247053342
O	4.6669212263	9.7899571625	6.1391943443
O	12.8571220551	6.4477838760	6.4643176208
O	4.6714267777	6.4043494329	6.4441741699
O	8.8909099304	12.9210117556	6.5326544931
O	0.6402900075	12.9221002996	6.5097343555
O	6.4355432934	12.3372250853	7.5067562979
O	14.6712538955	12.3434766621	7.5714669915
O	7.0283960553	2.7788833977	7.2116131820
O	10.8211891543	9.2293716104	7.5738215813
O	2.6306431244	9.2246651660	7.5793901226
O	15.2318119280	2.8333549187	7.2039695247
O	0.0611839483	10.1420979800	7.6635057532
O	8.2430957953	10.1368414027	7.6516678181
O	12.5494672971	3.9902042486	7.6456731958
O	4.3089939289	3.9644728419	7.6422437705
O	2.6276294337	6.1727078617	7.8696029079
O	10.8146833021	6.1687561140	7.8715938764
O	4.7280067926	1.2629895879	7.7943555753
O	12.9708638858	1.2894748941	7.8519931447
O	0.0987096631	5.2207878705	8.0840002538
O	8.2841501694	5.2101715624	8.0834083360
O	11.9853933718	11.6190159908	8.0868699723
O	3.7435990928	11.6136047865	8.0857310528
O	0.5479256907	7.7646917205	8.2339752253
O	8.7304542989	7.7578801344	8.2279174835
O	1.4003345936	2.6805405054	8.6033877907
O	9.6436935690	2.6871151343	8.6019089200
O	13.2956692693	9.2907825302	8.6647785003
O	5.0988150651	9.2918826840	8.6689572460
O	5.0320735756	6.1799454292	8.9910326138
O	13.2169124439	6.1788648876	9.0281323633
O	0.7896962859	12.2730971520	9.2452263480
O	8.9651608460	12.2725961133	9.2487796394
O	9.6911717145	9.4407922045	10.0013068625
O	1.5079563789	9.4371326440	10.0314281420
O	5.7659123095	2.9726183951	9.9018333151
O	14.0189367907	2.9844292564	9.9314942503
O	5.3034603077	12.9223698280	10.1247376266
O	13.4897466967	12.9165889924	10.1838418717
O	1.4294127341	6.3320692099	10.2695931608
O	9.6160916231	6.3307034294	10.2666698263
O	11.1415862409	4.0632293368	10.7100634048
O	2.9530779990	4.0609493420	10.7072699554
O	5.9408017013	7.8431790836	10.7782813940
O	14.1715439155	7.8522658320	10.7765481771
O	14.6377480063	10.3936505996	10.8415932535

O	6.4525246097	10.3967266723	10.7868211153
O	0.3251568260	3.7112011526	11.0793003485
O	8.5117743554	3.7036308487	11.0712775492
O	2.2750857739	1.3167921799	10.9252168779
O	10.4693788862	1.3165892004	10.9330196793
O	12.0902732771	9.4358483644	11.0805617941
O	3.9067238311	9.4402371547	11.1359327900
O	6.3506734228	5.3928532726	11.2979315111
O	14.5874265074	5.4280632329	11.3462664210
O	10.5165005643	11.6617892961	11.4418565408
O	2.3273339606	11.6700010458	11.4428130694
O	3.8298917482	6.4506606473	11.4323392754
O	12.0307717467	6.4544385484	11.4365670387
O	16.0778876837	12.7830312223	11.7902665669
O	7.8472604620	12.7845994128	11.7979651137
O	10.0424587022	9.2292983846	12.5533647209
O	1.8471530784	9.2199459044	12.5876518674
O	6.2639817492	2.6173820602	12.5469676635
O	14.5204872073	2.6204162724	12.5938817748
O	1.7951354669	5.8043516070	12.7518877104
O	9.9771324175	5.8059929687	12.7358826524
O	5.3618644990	12.4800417612	12.7425220871
O	13.6102226248	12.4812032705	12.8100840252
O	3.7736595150	2.3272724668	13.6204414586
O	12.0296027017	2.3186205353	13.6224580111
O	16.1179284768	8.6532678879	13.7747613260
O	7.9147671659	8.6436279333	13.7594958354
O	5.3425029511	9.6500587614	13.8343122865
O	13.5242279257	9.6735062630	13.9059332176
O	1.1855952790	3.4033167571	14.1291212974
O	7.8580668811	5.5931364726	14.0575644001
O	16.0580544080	5.6471206659	13.9868964923
O	9.4328315385	3.3832040747	14.0605526847
O	3.2473241824	11.3337047618	14.3507504535
O	11.4868452434	11.4704109779	14.5014560471
O	5.8386955548	7.2067917589	14.2832343290
O	14.0242804488	7.2435858358	14.2855158536
O	5.2487065252	4.7136129013	14.4149288672
O	9.7219248009	0.6676442195	14.5821136478
O	1.5411837801	0.6563489864	14.4206953688
O	13.4950385475	4.7098375005	14.4163850825
O	0.5619320900	11.0256050439	14.4825006122
O	8.8184185391	10.9906725258	14.5685974896
O	10.2720101462	8.7155485037	15.0158775265
O	2.0720109043	8.7818233862	15.0777841101
O	6.1832632464	2.1148670203	15.2212842257
O	14.4375525068	2.0989961747	15.2817453259
O	2.0006313691	5.6724361983	15.2954570210
O	10.3224262812	5.6531462653	15.2385452797
O	6.0789145949	12.0429580131	15.3004814530
O	14.3394110759	12.0560886614	15.3801371127
O	14.8414193497	8.9307973131	16.1726108404
O	6.5930694906	8.9311908919	16.1305172129

O	2.7259107979	2.3235280434	16.3875161306
O	10.9817504713	2.4758816836	16.3357337305
O	6.6583062363	5.8162548848	16.4551246179
O	14.9189401529	5.8829371411	16.4060753674
O	10.1414668885	12.3496476719	16.9026027563
O	1.9731831410	12.2761468302	16.6824116163
O	2.9064911767	7.3478173071	17.0555601633
O	11.1637651160	7.3407906831	16.9860273600
O	16.1405137334	3.5196634443	17.1214696400
O	7.8760989138	3.4663095890	17.1304234136
O	3.2697514551	9.8595810355	17.4185831771
O	11.4590891417	9.8745428042	17.2677600066
O	8.9479873126	8.8674480247	17.3382872713
O	0.7588823520	8.9316475099	17.4005628163
O	7.3606905499	0.7230783697	17.3390500843
O	15.6756237605	0.7265072797	17.4832376567
O	3.3917629035	4.9407583469	17.5580832807
O	11.7063619966	4.9561257466	17.5585807962
O	0.8092420641	5.8188352885	17.7081822163
O	9.0980383775	5.7367094326	17.6230284112
O	7.2613927135	10.9639824747	17.7761373951
O	15.4272065168	10.9414475457	17.8558616624
O	5.2459989501	2.8250093117	17.9076888669
O	13.5919147503	2.9119341509	17.9272789464
O	4.7358938242	12.2601261564	17.9275693455
O	12.9269548854	12.3519874858	17.9252357638
O	6.6570717155	8.4270546704	18.5926928752
O	14.9860607076	8.3545498643	18.6587320919
O	6.8152910619	5.2985673422	18.9415012050
O	14.8338546244	5.1580982782	18.8677974646
O	2.6933309534	2.5531561782	19.1449400003
O	11.0367442519	2.4593153377	19.0858631829
O	10.8745038057	11.5363358977	19.5926748653
O	2.2464426824	12.1314210559	19.2485360524
Ca	2.7291354393	7.8349644369	5.5578975726
Ca	10.9461264475	7.8384048369	5.5411627287
Ca	15.3024377892	0.9467477939	5.7556488019
Ca	7.1036939007	0.9399479445	5.7307604210
Ca	10.8715820254	1.5200966363	7.0123027870
Ca	2.6278535605	1.5035866526	7.0000361449
Ca	14.9308865972	8.4071928025	6.9703864221
Ca	6.6957095816	8.3783349204	6.9474018187
Ca	4.4658827149	1.3055312899	11.5532042701
Ca	12.6881153381	1.3353706814	11.5652562313
Ca	-0.2407920171	7.0138813012	11.9537680254
Ca	7.9472301208	6.9533474559	11.9637232835
Ca	3.7161484537	7.9633970097	13.4094462245
Ca	11.9332782965	7.9718986748	13.4057777030
Ca	7.5000237379	0.6512815775	13.8343183535
Ca	15.7446819071	0.6436336443	13.8456485207
Ca	1.4626209316	0.8228433088	17.9878312258
Ca	9.5113683464	1.1866495583	17.6942012751
Ca	5.0208663540	6.6177685788	18.3976005678

Ca	13.2383583091	6.7557979003	18.3806903899
H	5.4830237331	2.8391111901	18.8949199560
H	13.9852470840	3.6614545858	18.4844378342
H	6.7242654201	8.6307434552	19.5661031602
H	15.5929425610	7.9617095282	19.3936376769
H	2.2823962435	3.2510003980	19.7068055579
H	11.7041754725	1.9384643970	19.6096219798
H	2.7684640698	12.2260409678	20.0642091428
H	11.3796005703	12.2324587091	20.0771373872
O	5.0960476287	11.9855684889	20.6122827314
O	12.9518846977	7.0520090792	20.6917321335
O	16.1647781738	6.9737738150	20.5319913357
O	8.3718329568	12.2722186192	19.9613578071
O	12.5648037718	13.7226058661	20.1051310963
O	5.8284033561	2.8277863834	20.4737803666
O	8.1537388113	6.1847946490	20.8489342265
O	4.5328515182	5.5112585329	20.6222980308
O	8.3404106129	1.8262129818	20.0367472297
O	10.4540269305	7.4003072193	19.8974797387
O	12.8944558661	9.7274863156	20.1912232875
O	3.8745758064	8.3488437698	19.6712181818
O	1.8310731355	4.8642661468	20.6212043664
O	6.4916463327	8.2043633794	21.2690806105
H	5.0538770527	12.1234511400	19.6173795019
H	6.0502474253	12.0029679699	20.8076567077
H	13.1377575493	8.0244533095	20.7892313445
H	11.9580782477	7.0605778095	20.7257814805
H	15.6478377344	6.1871893728	20.2102042146
H	17.0840350165	6.6855474655	20.3521165449
H	7.9383369572	11.8049003607	19.2002770721
H	9.3287037026	11.9340247810	19.9239958933
H	13.3559877145	13.8535456850	20.6523362319
H	12.8745443776	13.2615890631	19.2374845637
H	5.3419489413	2.0402731645	20.7909777830
H	6.7863762230	2.5286511248	20.4187422313
H	8.3724233805	5.4811074579	21.4836382669
H	7.6185673690	5.7372269293	20.0499191976
H	4.8974317449	6.0915309373	21.3197027050
H	5.0835624864	4.6850848935	20.6466807448
H	9.1958854018	2.2241838427	20.3005255979
H	8.4279799189	0.8273146239	20.1744449814
H	9.6011391798	6.9624361711	20.1634019282
H	10.2625832641	8.3525514527	19.8328566551
H	12.1170803612	10.3370818886	20.0901265328
H	13.6129640835	10.1407921920	19.6781178101
H	4.3770024833	8.8393456204	20.3496665570
H	3.6258368657	9.0349600137	18.9945583838
H	1.4690302999	4.7177662263	21.5128774483
H	2.7676538438	5.1460437825	20.7635124065
H	7.1793224308	7.4660418228	21.2930779915
H	6.7538231809	8.8555116388	21.9429289929

B. Anorthite slab + 9 CO_2

```
CRYSTAL
 PRIMVEC
   16.416934674    0.000000000    0.000000000
   -0.357421897   13.093383045    0.000000000
   -0.317241471   -0.058933559   34.635155472
 PRIMCOORD          1
        343          1
Si       6.838721155   10.629329065    1.643193293
Si      15.047401215   10.567735691    1.650344375
Si       4.184326938    9.818117026    1.889021101
Si      12.409108953    9.809546454    1.842853622
Si       6.626995499    5.570251661    2.212339066
Si      14.819718605    5.549379101    2.235271696
Si      12.164232988    6.861666679    2.283538800
Si       3.975220307    6.879808215    2.283640817
Si       0.064368079    9.865073120    4.194374387
Si       8.293689066    9.905873787    4.160921904
Si       8.130316724    6.775878030    4.366314726
Si      -0.107591455    6.746378913    4.394576022
Si       5.317853837   10.723306754    4.831663044
Si      13.519308127   10.722034071    4.812149454
Si       5.077981139    5.835295638    4.835003730
Si      13.269907570    5.850828579    4.859670110
Si      12.176008798   10.147879325    7.588745665
Si       3.991965020   10.145315873    7.620773936
Si       4.124869461    5.496597107    7.724694283
Si      12.302934249    5.529734784    7.723072643
Si       9.329910132    9.305675870    8.373670619
Si       1.154518096    9.304407169    8.374616044
Si       1.137898777    6.236633936    8.544519693
Si       9.313980825    6.239326286    8.555172010
Si      13.503963339    9.364848845   10.270628658
Si       5.321833677    9.358611035   10.289191942
Si       5.223603397    6.308476722   10.543866159
Si      13.431100611    6.318832910   10.543945362
Si      10.526578726   10.125739128   11.169448658
Si       2.350496800   10.132613116   11.172701068
Si       2.440395129    5.434429313   11.208180230
Si      10.634561998    5.447657461   11.218648805
Si       9.250097566    9.600615866   13.957572417
Si       1.069074013    9.602048202   13.960561419
Si       1.135996422    4.948355020   14.024157656
Si       9.340770935    4.937879517   14.039880053
Si       6.423269699    8.760346353   14.503666566
Si      14.655448521    8.774171415   14.493951432
Si       6.365349130    5.712671207   14.746232058
Si      14.585816952    5.746180968   14.713576988
Si      10.361336255    8.824641690   16.704636050
Si       2.192636590    8.818871615   16.704105862
Si       2.176669323    5.809860283   16.875476642
```

Si	10.360319707	5.807554578	16.905658798
Si	7.351862287	9.473018824	17.425482473
Si	15.607880775	9.493627503	17.411175894
Si	15.735722999	5.039884343	17.540623264
Si	7.533891314	4.969035055	17.579523771
Al	8.454206839	3.260526138	1.568419566
Al	0.265515278	3.275147955	1.491576157
Al	2.890413993	12.514837932	1.582201566
Al	11.066962452	12.506096382	1.580102593
Al	10.832732008	4.108827103	1.721492031
Al	2.654663130	4.117797696	1.673030562
Al	8.331061903	0.294451477	2.176483833
Al	0.139527322	0.293706243	2.141526627
Al	13.035341280	3.364526560	3.656406088
Al	4.827746601	3.347820047	3.622641105
Al	12.699509921	0.464328819	4.032358566
Al	4.501459420	0.451650629	4.040656283
Al	10.088524330	4.317928130	4.669349770
Al	1.881349248	4.281342428	4.624248582
Al	9.348982026	12.560509251	4.848359950
Al	1.132321615	12.549236042	4.823203610
Al	8.559952029	3.513180528	7.459621466
Al	0.346318261	3.521850140	7.402266980
Al	-0.130612267	12.000567803	7.759751189
Al	8.052271466	12.008438430	7.784663124
Al	5.537039232	2.752982813	8.180646850
Al	13.758283995	2.794709208	8.168997460
Al	4.987520223	12.841920068	8.410643787
Al	13.210628420	12.860084921	8.391391562
Al	1.604937120	2.810092722	10.249552675
Al	9.788422312	2.809402286	10.308870182
Al	1.060884538	12.857614467	10.744769319
Al	9.239595585	12.859160364	10.785086506
Al	6.769392493	3.538813684	11.147304488
Al	15.010055918	3.554488150	11.135477303
Al	14.471430615	12.136128177	11.240009831
Al	6.260308681	12.125020689	11.264688743
Al	5.303768816	2.948620080	13.987173980
Al	13.545732900	2.949471542	13.981168201
Al	4.886807684	11.488413916	14.105001894
Al	13.092752478	11.500011696	14.087201970
Al	2.347296187	2.150289068	14.587988316
Al	10.579111762	2.141805071	14.585405735
Al	1.835369794	12.200364532	14.894886423
Al	10.050853848	12.202411408	14.877663142
Al	6.640319841	2.183895150	16.866967171
Al	14.853470716	2.213251032	16.864796206
Al	6.140200556	12.266079820	17.055942003
Al	14.356579189	12.277973769	17.047965750
Al	3.472328640	3.113869078	17.609130914
Al	11.693133322	3.135494618	17.612438939
Al	3.101695911	11.599637329	17.767044164
Al	11.325638024	11.600808981	17.765517027

O	9.770557167	3.560356930	0.502343186
O	1.580771067	3.631366157	0.435459637
O	13.875950423	9.679855049	1.015929884
O	5.651992909	9.729114323	1.073427174
O	11.385664026	10.833341789	1.213179704
O	3.191977476	10.841780736	1.219110436
O	7.155455342	4.442827844	1.292348800
O	15.342553855	4.434667026	1.298570833
O	1.663382354	-0.140475975	1.448495361
O	9.839186516	-0.141602491	1.448991845
O	6.880009164	12.136940010	1.358722823
O	15.127567923	12.073883813	1.368723443
O	15.902211191	1.811796280	1.815535731
O	7.655258329	1.822147310	1.957237185
O	5.444270792	6.543161145	1.652257579
O	13.619610222	6.481563772	1.653962221
O	3.477921606	8.369903603	1.877924504
O	11.709045892	8.362732994	1.872673081
O	10.984955876	5.873332936	2.008040351
O	2.815006375	5.871597705	2.005602278
O	12.303183266	3.341705244	2.154991896
O	4.126405945	3.338900435	2.099370543
O	12.741766251	0.063949918	2.376724178
O	4.568506206	0.067728050	2.380271819
O	-0.363514973	9.747947458	2.599298311
O	7.883079321	9.822570309	2.558944765
O	7.818762313	6.550210949	2.809166617
O	-0.424238975	6.555173458	2.829184566
O	9.578877951	3.785882212	3.096365433
O	1.401429057	3.778475830	3.032938559
O	12.976231812	10.108202262	3.397897098
O	4.711441734	10.101898875	3.454136838
O	14.193531655	4.865188765	3.740005427
O	5.966925093	4.866676990	3.688821561
O	8.293302074	-0.156138793	3.899127914
O	0.101760006	-0.086525909	3.883981144
O	12.483272366	7.047937597	3.967302235
O	4.293331798	7.051345103	3.970497865
O	1.331727564	10.754289500	4.551181611
O	9.575943061	10.764243508	4.557142976
O	8.699548482	8.350949852	4.634620821
O	0.463108979	8.316552954	4.699410069
O	13.547470575	1.947494394	4.486089541
O	5.300557490	1.954900170	4.531470519
O	11.154476243	0.421863472	4.830267348
O	2.955365680	0.363839914	4.822177869
O	5.160809330	12.288835243	4.919267053
O	13.339432992	12.284934359	4.915832532
O	3.837558904	4.563791143	4.753161363
O	12.035421515	4.573460660	4.765314950
O	6.863306942	10.107818211	4.991276560
O	15.064401705	10.111720036	5.041845750
O	1.194493719	5.963248285	4.978411939

O	9.434050735	6.020539238	4.979975281
O	6.703094256	6.686508888	5.210702835
O	14.888847512	6.682205797	5.261449747
O	9.559106759	3.335060604	5.993269476
O	1.322977565	3.277770885	5.925032132
O	12.801313929	9.796958400	6.065720643
O	4.636821532	9.805416689	6.109231628
O	12.812534826	6.448695918	6.412692877
O	4.649497079	6.393783323	6.407747392
O	8.790101284	12.937021148	6.482025078
O	0.599604362	12.931756542	6.458896291
O	6.377366810	12.342682680	7.498065179
O	14.611903148	12.350407697	7.499024566
O	7.006090980	2.760981003	7.206150061
O	10.771772891	9.235395774	7.510092777
O	2.594080868	9.227418310	7.511082437
O	15.187252329	2.825481770	7.142091249
O	0.018783392	10.125958909	7.591408484
O	8.199388858	10.139732646	7.595109694
O	12.516457665	3.982844518	7.576995837
O	4.286661917	3.940809743	7.582511412
O	2.602591385	6.165822485	7.795684427
O	10.774374791	6.165498575	7.805338440
O	4.706979964	1.242723137	7.783149193
O	12.940302347	1.278395925	7.780155031
O	0.077546309	5.208743402	8.003878258
O	8.252158977	5.208788035	8.014842588
O	11.926696959	11.628187058	8.011006852
O	3.695337399	11.609781359	8.076371714
O	0.520539751	7.754257777	8.158503882
O	8.694287120	7.756702292	8.160030942
O	1.386599130	2.676440744	8.520970372
O	9.617876889	2.679861702	8.577179939
O	13.239891677	9.294138028	8.592480840
O	5.064885955	9.288929635	8.611532500
O	4.998036622	6.168268779	8.949616208
O	13.175486635	6.174568874	8.956750742
O	0.748962090	12.274802833	9.165374601
O	8.916247433	12.280326621	9.202919235
O	9.644669831	9.439133172	9.952694940
O	1.468616872	9.439813837	9.954925608
O	5.737988110	2.962500792	9.885510107
O	13.977884635	2.967939741	9.879194276
O	5.248642601	12.929589889	10.103891473
O	13.425346407	12.927626762	10.096683147
O	1.406940059	6.320582681	10.195296357
O	9.583019222	6.321815491	10.211718469
O	11.109339348	4.053538475	10.678961138
O	2.929385210	4.046959482	10.665770699
O	5.901541369	7.842008638	10.737938724
O	14.125583189	7.852825566	10.684896328
O	14.584476081	10.401136120	10.744629295
O	6.407127190	10.395783984	10.750992195

O	0.302753942	3.700204492	10.983797657
O	8.480434555	3.692751619	11.038446973
O	2.266366701	1.299686838	10.827712764
O	10.444087797	1.299869119	10.895695236
O	12.044215732	9.435641847	11.039523343
O	3.860653179	9.440108376	11.042732205
O	6.320836790	5.393279026	11.261663020
O	14.555940566	5.426271648	11.257264146
O	10.457655032	11.666203346	11.394828539
O	2.277490717	11.675935348	11.394147161
O	3.797586634	6.443021582	11.384418271
O	11.999848226	6.448120262	11.389585533
O	16.015784781	12.792627795	11.695145398
O	7.788517254	12.788281991	11.754802720
O	9.991234418	9.211944833	12.495500298
O	1.814091948	9.215558737	12.499283288
O	6.245148400	2.602036195	12.544928324
O	14.488250938	2.612093168	12.534935779
O	1.768991157	5.798434428	12.692840079
O	9.951004458	5.800484411	12.701695260
O	5.308052668	12.476235365	12.712234076
O	13.549948932	12.486081316	12.706138916
O	3.751628321	2.305611128	13.573635611
O	11.992550017	2.301574488	13.576405710
O	16.110582731	8.631466033	13.710458731
O	7.877275318	8.634806329	13.713801176
O	5.304139376	9.653244705	13.783627964
O	13.541082793	9.673137029	13.779810592
O	1.162514746	3.388014916	14.005127044
O	7.802741249	5.578165233	13.924769770
O	16.031529620	5.638041329	13.910204362
O	9.407472534	3.381395219	14.004726857
O	3.188297121	11.316684711	14.277619474
O	11.387683858	11.319212424	14.215410239
O	5.793353052	7.214279849	14.219072044
O	13.978200127	7.242423758	14.215175989
O	5.217196721	4.699899407	14.360969798
O	9.713474643	0.633480351	14.362347367
O	1.518942823	0.628409835	14.355518629
O	13.460155303	4.701472357	14.359118156
O	0.525320003	10.996697947	14.433183809
O	8.745707281	11.006201075	14.431918555
O	10.219089630	8.716954664	15.008479841
O	2.037297067	8.714981076	15.008576794
O	6.155482505	2.084578297	15.225390644
O	14.397452343	2.086012961	15.218950482
O	1.960479833	5.646747549	15.257925071
O	10.131661674	5.644785433	15.290341328
O	6.012232455	12.052132934	15.306018930
O	14.196407003	12.055950205	15.307330736
O	14.780808795	8.913257716	16.092329658
O	6.535718874	8.910737314	16.097783554
O	2.633507806	2.374222903	16.317022630

O	10.877864744	2.374326008	16.316371976
O	6.693738750	5.867844712	16.340185830
O	14.928125584	5.940099496	16.301001864
O	10.239400862	12.332895566	16.599174351
O	1.996305316	12.332458081	16.615775836
O	2.865349065	7.321179661	17.033746830
O	11.040021536	7.327456194	17.038796650
O	16.021319442	3.531820174	17.100187770
O	7.848218799	3.472637205	17.100542233
O	3.211569327	9.853484102	17.317294310
O	11.393198936	9.850069712	17.317397351
O	8.860986878	8.845851842	17.315790815
O	0.685384497	8.844832666	17.315678248
O	7.390300600	0.702327627	17.387544975
O	15.579297395	0.723112300	17.391357507
O	3.360526178	4.918537708	17.530230397
O	11.585413218	4.940297138	17.529012767
O	0.775492245	5.794033505	17.690235056
O	8.969690841	5.777752882	17.743109610
O	7.282255656	11.018274477	17.680399664
O	15.519344371	11.035705697	17.675511764
O	5.247785819	2.880312780	17.889400105
O	13.473624408	2.939359841	17.885312863
O	4.682563022	12.198778048	17.961035236
O	12.913256273	12.186890622	17.968114188
O	6.533273143	8.555446218	18.599737365
O	14.766419528	8.624391317	18.613458723
O	6.469548746	5.137255652	18.762504161
O	14.646652215	5.275909723	18.696463049
O	2.670014304	2.436381290	19.043415396
O	10.909154043	2.435696934	19.046235752
O	10.298892377	12.056092733	19.187009873
O	2.053460890	12.047490454	19.177442445
Ca	2.678823496	7.755956899	5.550384399
Ca	10.893199954	7.836997428	5.509880147
Ca	15.294876282	0.948606334	5.694906859
Ca	7.071811945	0.931826107	5.711581788
Ca	10.827371837	1.501702311	6.974691224
Ca	2.616822862	1.484841941	6.942805381
Ca	14.867431329	8.400655419	6.910570210
Ca	6.652530202	8.390709859	6.886753287
Ca	4.449334794	1.292370476	11.517252646
Ca	12.660714746	1.314117986	11.503367703
Ca	-0.251726072	7.016201638	11.862278415
Ca	7.925379785	6.973414846	11.901815024
Ca	3.700621115	7.987903453	13.311632770
Ca	11.906318566	8.048942441	13.302622546
Ca	7.470936440	0.618514277	13.804579037
Ca	15.698486241	0.615801950	13.778219203
Ca	1.376936387	0.752500250	17.902470320
Ca	9.610847043	0.762823786	17.869040764
Ca	5.077181704	6.708592057	18.032330643
Ca	13.281967089	6.819288190	17.901582134

H	5.642187354	3.662665377	18.402890584
H	13.843765832	3.761414769	18.339426185
H	6.712223448	8.905638589	19.494640353
H	14.566102072	9.229407884	19.353095657
H	3.241269517	2.190694507	19.787783824
H	11.488077652	2.212669266	19.793335191
H	2.405775962	11.975163361	20.077037443
H	10.766136821	12.186979752	20.028048728
C	14.622990541	4.045405112	21.051582632
C	9.631431569	3.463877260	21.814551965
C	3.767758049	6.658136244	21.505295070
C	7.569618072	10.728157447	22.007503458
C	9.938137887	7.778775319	22.869982501
C	13.898086849	7.089573566	22.985501510
C	15.884362061	1.011257854	23.092508828
C	3.278773455	11.022719180	23.166411464
C	12.030219931	0.467908254	23.305088012
O	15.752647434	3.752228585	20.931634652
O	7.626048839	10.132647393	20.993963418
O	8.878476064	4.187871630	21.282807496
O	13.486687824	4.315180897	21.209263243
O	4.223517386	6.592669960	20.419594375
O	10.478739076	6.866580132	22.365855246
O	2.486000484	11.019954987	22.295689279
O	3.328674578	6.726302600	22.583940654
O	14.993664658	1.408512089	22.438087690
O	10.373760241	2.734661471	22.363830334
O	13.290839264	8.040473084	22.651990911
O	11.473466823	0.025910250	22.367428107
O	7.506172281	11.316379781	23.017673527
O	14.508939014	6.144680327	23.314642140
O	9.396840528	8.689960336	23.377245223
O	16.775424456	0.610991242	23.742341079
O	4.075312864	11.015415967	24.027246423
O	12.587408203	0.901606434	24.242121051
